# Galectin-3 Contributes to the Inhibitory Effect of lα,25-(OH)_2_D_3_ on Osteoclastogenesis

**DOI:** 10.3390/ijms222413334

**Published:** 2021-12-11

**Authors:** Jianhong Gu, Xueqing Zhang, Chuang Zhang, Yawen Li, Jianchun Bian, Xuezhong Liu, Yan Yuan, Hui Zou, Xishuai Tong, Zongping Liu

**Affiliations:** 1 College of Veterinary Medicine, Yangzhou University, Yangzhou 225009, China; jhgu@yzu.edu.cn (J.G.); MX120190776@yzu.edu.cn (X.Z.); zc025032@outlook.com (C.Z.); MX120200961@yzu.edu.cn (Y.L.); jcbian@yzu.edu.cn (J.B.); xjliu@yzu.edu.cn (X.L.); yuanyan@yzu.edu.cn (Y.Y.); zouhui@yzu.edu.cn (H.Z.); 2 Jiangsu Co-Innovation Center for Prevention and Control of Important Animal Infectious Diseases and Zoonoses, Yangzhou 225009, China; 3 Dafeng Port Economic Development Zone Administrative Committee in Jiangsu, Yancheng 224145, China; 4 Jiangsu Key Laboratory of Zoonosis, Yangzhou 225009, China; 5 Joint International Research Laboratory of Agriculture and Agri-Product Safety of the Ministry of Education of China, Institutes of Agricultural Science and Technology Development, Yangzhou University, Yangzhou 225009, China

**Keywords:** galectin-3, lα,25-(OH)_2_D_3_, osteoclasts, bone resorption, siRNA

## Abstract

The active form of vitamin D, 1α,25-(OH)_2_D_3_, not only promotes intestinal calcium absorption, but also regulates the formation of osteoclasts (OCs) and their capacity for bone mineral dissolution. Gal-3 is a newly discovered bone metabolic regulator involved in the proliferation, differentiation, and apoptosis of various cells. However, the role of galectin-3 (gal-3) in OC formation and the regulatory effects of 1α,25-(OH)_2_D_3_ have yet to be explored. To confirm whether gal-3 contributes to the regulatory effects of 1α,25-(OH)_2_D_3_ on osteoclastogenesis, osteoclast precursors (OCPs) were induced by macrophage colony stimulating factor (M-CSF) and receptor activator of nuclear factor κB ligand (RANKL). TRAP staining and bone resorption analyses were used to verify the formation and activation of OCs. qPCR, Western blotting, co-immunoprecipitation, and immunofluorescence assays were used to detect gene and protein expression. The regulatory effects of gal-3 in OC formation after treatment with 1α,25-(OH)_2_D_3_ were evaluated using gal-3 siRNA. The results showed that 1α,25-(OH)_2_D_3_ significantly increased gal-3 expression and inhibited OC formation and bone resorption. Expression levels of OC-related genes and proteins, matrix metalloproteinase 9 (MMP-9), nuclear factor of activated T cells 1 (NFATc1), and cathepsin K (Ctsk) were also inhibited by 1α,25-(OH)_2_D_3_. Gal-3 knockdown attenuated the inhibitory effects of 1α,25-(OH)_2_D_3_ on OC formation, activation, and gene and protein expression. In addition, gal-3 was co-localized with the vitamin D receptor (VDR). These data suggest that gal-3 contributes to the osteoclastogenesis inhibitory effect of lα,25-(OH)_2_D_3_, which is involved in bone and calcium homeostasis.

## 1. Introduction

OCs are derived from bone marrow mononuclear macrophages (BMMs) and are the only cells capable of bone resorption in the body. They not only degrade the bone organic and inorganic matrix but also cooperate with osteoblasts (OBs) to regulate bone formation and reconstruction [1,2,3]. Overactive bone resorption by OCs in physiological processes (such as aging and menopause) [4] and pathological processes (such as bone metastasis and rheumatoid arthritis) [5,6] can lead to osteoporosis. OCPs, such as bone marrow cells, splenocytes, and RAW264.7 macrophages, co-cultured with stromal cells, OBs, or osteocytes in vitro can be induced into OCs by the parathyroid hormone (PTH), dexamethasone, tumor necrosis factor-alpha (TNF-α), interleukin-1 beta (IL-1β), and 1α,25-(OH)_2_D_3_, which regulate the expression of membrane-bound RANKL in OBs, stromal cells, and osteocytes [1,7]. BMMs could also be induced into OCs directly by M-CSF and RANKL [8]. M-CSF mainly promotes the proliferation and differentiation of OCPs, which further differentiate into OCs under the action of RANKL [9,10].

The physiologically active form of vitamin D, 1α,25-(OH)_2_D_3_, regulates intestinal calcium absorption and acts on bone cells directly and a series of cytokines or signaling pathways in bone [11,12,13]. OB-lineage cells express vitamin D receptor (VDR) [14], and 1α,25-(OH)_2_D_3_ promotes OBs’ maturation and bone mineralization in vitro and in vivo via VDR and reduces the formation of unmineralized osteoids [15]. However, bone tissue is in a state of dynamic equilibrium, and over-mineralization or absorption is not conducive to bone health. To prevent excessive bone mineralization, 1α,25-(OH_)2_D_3_ can enhance OC formation indirectly by promoting the expression of RANKL in a concentration-dependent manner [15,16]. OC formation can also be directly regulated by 1α,25-(OH)_2_D_3_. Although mature OCs do not express VDR, OCPs do [17,18,19]. The mechanism by which it regulates OC formation needs to be further clarified.

Gal-3 is a 29–35 kDa protein expressed in a variety of tissues and is a member of the β-galactosyl-binding protein family [20]. It is a marker of chondrocyte and OB lineages in bone and is also present in OCs and BMMs [21,22]. In proteomic studies, we found that 1α,25-(OH)_2_D_3_ activates gal-3 expression during OC formation in vitro [23,24]. Simon et al. have also shown that gal-3 is a novel regulator of bone homeostasis directly or indirectly by regulating the association between OBs and OCs. Accordingly, gal-3 plays an important role in bone biology and is expected to be a potential target for the prevention of bone diseases. However, its role in the regulation of OC formation by 1α,25-(OH)_2_D_3_ needs to be further elucidated.

In this study, M-CSF and RANKL were used to induce osteoclastogenesis in mouse OCPs in vitro. We aimed to investigate the role of gal-3 in osteoclastogenesis after treatment with 1α,25-(OH)_2_D_3_. Our results provide insights into the mechanism underlying the regulation of osteoclastogenesis by 1α,25-(OH)_2_D_3_.

## 2. Results

### 2.1. 1α,25-(OH)_2_D_3_ Had No Effect on Osteoclast Precursor Viability

**Figure 1:**
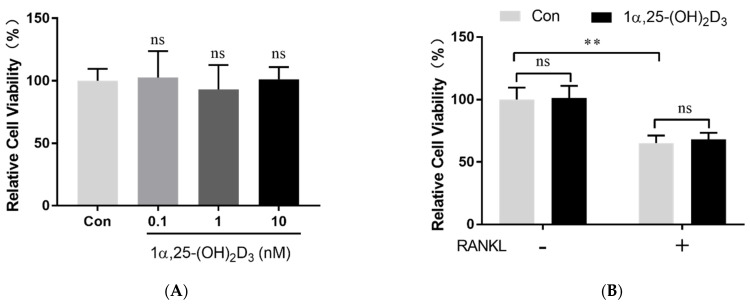
OCP viability was not affected by 1α,25-(OH)_2_D_3_. (**A**) Cell viability detected by CCK-8 24 h after treatment with different concentrations of 1α,25-(OH)_2_D_3_. (**B**) Cell viability detected by CCK-8 24 h after treatment with 10 nmol/L 1α,25-(OH)_2_D_3_ in the absence or presence of 50 ng/mL RANKL. Data are shown as means ± SD. n = 6, ns, *p* > 0.05; ** *p* < 0.01.

We confirmed that 1α,25-(OH)_2_D_3_ upregulates VDR mRNA and protein expression in OCPs [25]. In this study, we observed that adding 0.1, 1, and 10 nmol/L 1α,25-(OH)_2_D_3_ to the medium had no effect on OCPs’ viability (Figure 1A). RANKL significantly inhibited cell proliferation during OCs formation (*p* < 0.01). However, 1α,25-(OH)_2_D_3_ had no significant effect on OCPs’ viability in the absence or presence of RANKL (Figure 1B).

### 2.2. 1α,25-(OH)_2_D_3_ Promoted Gal-3 Expression

**Figure 2:**
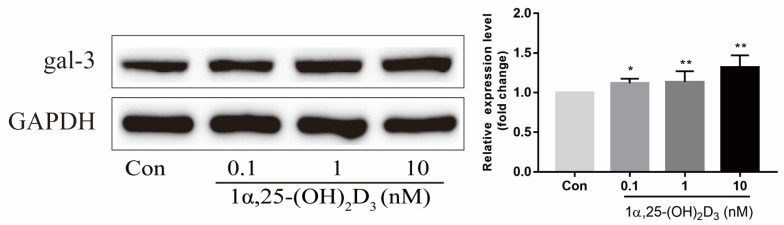
The expression of gal-3 protein was upregulated by 1α,25-(OH)_2_D_3_ dose-dependently on day 3 as determined by Western blotting. Histograms show gray values of gal-3 protein. Data are shown as means ± SD. n = 5, * *p* < 0.05, ** *p* < 0.01.

To elucidate the effect of 1α,25-(OH)_2_D_3_ on gal-3 protein expression, 0.1, 1, and 10 nmol/L 1α,25-(OH)_2_D_3_ were added to the culture medium during OC formation induced by 25 ng/mL M-CSF and 50 ng/mL RANKL for 3 days. 1α,25-(OH)_2_D_3_ upregulated gal-3 protein expression in a dose-dependent manner (Figure 2). The 10 nmol/L 1α,25-(OH)_2_D_3_ group had a higher level of gal-3 protein expression than that in the other groups.

**Figure 3:**
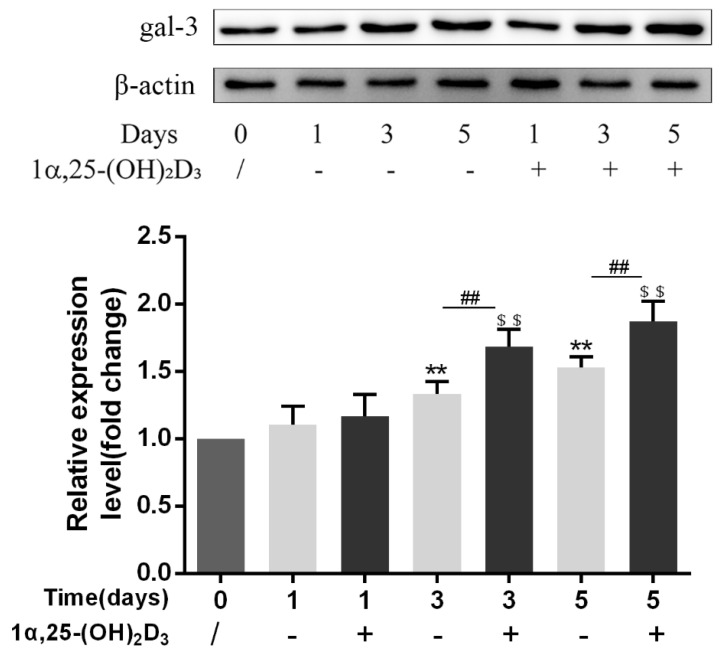
Gal-3 protein expression was upregulated by 1α,25-(OH)_2_D_3_ at the same cultivation time. Histograms show relative expression levels of gal-3 protein. Data are shown as means ± SD. n = 3, ** *p* < 0.01 vs. the 0 d group; ^$$^ *p* < 0.01 vs. the 1α,25-(OH)_2_D_3_ treatment group on day 1; ^##^ *p* < 0.01 vs. different groups on the same day.

To confirm the effect of 1α,25-(OH)_2_D_3_ on gal-3 protein expression at different time points, OCPs induced by 25 ng/mL M-CSF and 50 ng/mL RANKL were treated with 10 nmol/L 1α,25-(OH)_2_D_3_ for 0, 1, 3, and 5 days. Anhydrous ethanol was used as a control. Compared with the level in the control group, 1α,25-(OH)_2_D_3_ significantly increased gal-3 protein expression on days 3 and 5 (*p* < 0.01) (Figure 3). No significant difference was observed between the control group and the 1α,25-(OH)_2_D_3_ group on day 1 (*p* > 0.05). Compared with day 1, 10 nmol/L 1α,25-(OH)_2_D_3_ significantly increased gal-3 protein expression on days 3 and 5 (*p* < 0.01). However, there was no significant change between days 3 and 5 1α,25-(OH)_2_D_3_ groups (*p* > 0.05). These data indicated that 1α,25-(OH)_2_D_3_ promoted the protein expression of gal-3 at the same cultivation time.

**Figure 4:**
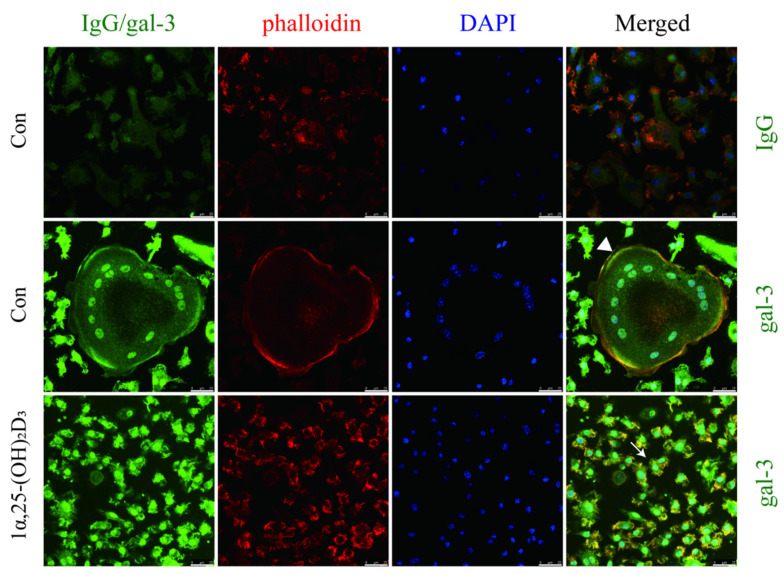
Immunofluorescence showed the distribution and expression of gal-3 after the treatment of 1α,25-(OH)_2_D_3_. Gal-3 mainly distributed in the nuclei (cyan) and cell membranes (yellow) of OCs (marked by white triangle) and in the whole cell of OCPs (white arrows). Gal-3 protein distribution was regulated by 1α,25-(OH)_2_D_3_. In non-merged images, green indicates gal-3, red indicates F-actin, and blue indicates nuclei. Bars = 25 μm.

To further confirm the effect of 1α,25-(OH)_2_D_3_ on gal-3 protein distribution, an immunofluorescence assay was performed. The gal-3 protein was visualized by green fluorescence, while F-actin was visualized in red on day 6 after treatment with 10 nmol/L 1α,25-(OH)_2_D_3_. Anhydrous ethanol was used as a control. Gal-3 mainly distributed in the nuclei (cyan) and cell membranes (yellow) of OCs (large cells with more than three nuclei marked by white triangles) and in the whole OCPs (small cells with one nucleus marked by white arrow) (Figure 4). Compared with OCs, OCPs had a wider green fluorescence distribution of gal-3. These data confirmed that 1α,25-(OH)_2_D_3_ changed the protein distribution of gal-3.

**Figure 5:**
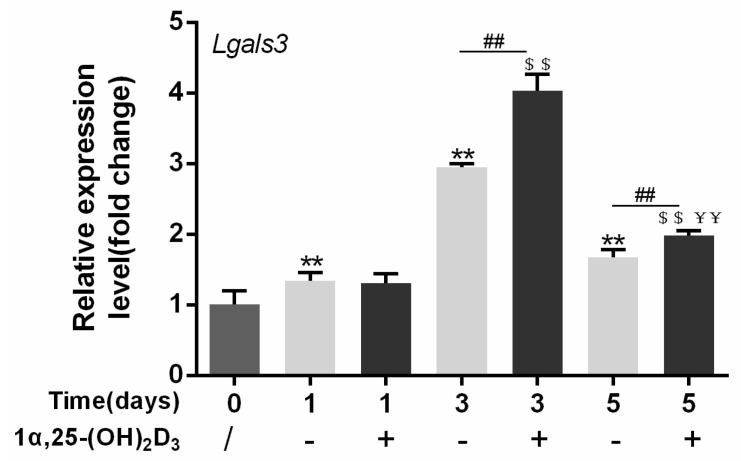
*Lgals3* expression is upregulated by 1α,25-(OH)_2_D_3_. Data are shown as means ± SD. n = 3, ** *p* < 0.01 vs. the 0 d group; ^$$^ *p* < 0.01 vs. the 1α,25-(OH)_2_D_3_ treatment group on day 1; ^##^ *p* < 0.01 vs. different groups on the same day; ^¥¥^ *p* < 0.01 vs. the 1α,25-(OH)_2_D_3_ treatment group on day 3.

To elucidate the effect of 1α,25-(OH)_2_D_3_ on the expression of *Lgals3,* which encodes the gal-3 protein, 10 nmol/L 1α,25-(OH)_2_D_3_ was added to the culture medium during OC formation induced by 25 ng/mL M-CSF and 50 ng/mL RANKL for 0, 1, 3, and 5 days. Anhydrous ethanol was used as a control. The expression of *Lgals3* first increased and then decreased over time. Compared with control groups (without 1α,25-(OH)_2_D_3_), 10 nmol/L 1α,25-(OH)_2_D_3_ significantly increased *Lgals3* expression on days 3 and 5 (*p* < 0.01). Compared with day 1, 10 nmol/L 1α,25-(OH)_2_D_3_ significantly increased *Lgals3* expression on days 3 and 5 (*p* < 0.01). However, *Lgals3* expression on day 5 was lower than day 3 in the groups with or without 1α,25-(OH)_2_D_3_ (Figure 5).

### 2.3. Gal-3 Contributed to Osteoclasts Formation and Activation Regulated by 1α,25-(OH)_2_D_3_

We found that 1α,25-(OH)_2_D_3_ increased gal-3 expression at the mRNA and protein levels. To confirm the role of gal-3 in 1α,25-(OH)_2_D_3_-mediated OC formation and bone resorption, we constructed stable *Lgals3* knockdown OCPs using gal-3 siRNA. Negative control (NC) siRNA was used as the control. These OCPs were treated with 10 nM 1α,25-(OH)_2_D_3_ in the presence of 25 ng/mL M-CSF and 50 ng/mL RANKL. Anhydrous ethanol was used as a control.

**Figure 6:**
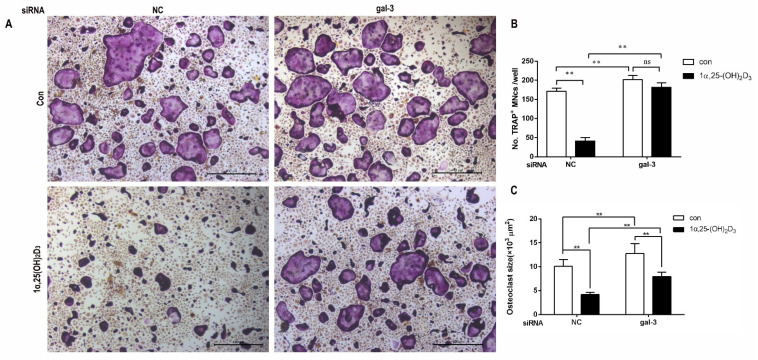
Gal-3 knockdown attenuated the inhibitory effect of 1α,25-(OH)_2_D_3_ on OC formation. (**A**) TRAP staining. Large multinuclear cells (MNCs) with wine-red granules were regarded as OCs (yellow arrows). Bars = 400 μm. (**B**) Quantitative analysis of OC quantity. (**C**) Quantitative analysis of OC size. Data are shown as means ± SD. n = 3, ** *p* < 0.01, ns means *p* > 0.05.

First, OC formation was detected by TRAP staining on day 6 after the treatment of 1α,25-(OH)_2_D_3_. In all groups, large cells with wine-red granules regarded as OCs were found. In the NC group, the volume of OCs treated with 1α,25-(OH)_2_D_3_ and the number and the size of OCs decreased significantly (*p* < 0.01). In the gal-3 knockdown group, 1α,25-(OH)_2_D_3_ had no significant effect on OC formation, but significantly decreased the size of OCs. These data confirmed that gal-3 contributes to the regulation of OC formation by 1α,25-(OH)_2_D_3_. Additionally, gal-3 knockdown significantly promoted OC formation and average size (*p* < 0.01) (Figure 6A–C). This suggests that gal-3 is a negative regulator of OC formation and average size.

**Figure 7:**
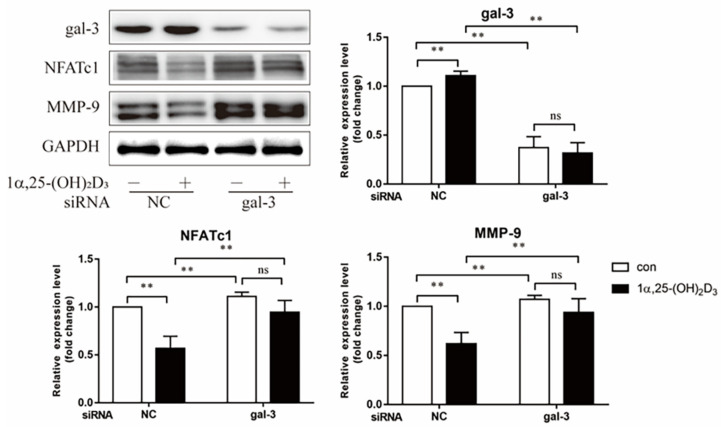
Gal-3 knockdown attenuated the inhibitory effect of 1α,25-(OH)_2_D_3_ on OC-related protein expression. The histograms show the relative expression level of proteins. Data are shown as means ± SD. n =3, ** *p* < 0.01, ns means *p* > 0.05.

Gal-3 and OC-related proteins (NFATc1 and MMP-9) were investigated by Western blotting on day 3 after treatment with 1α,25-(OH)_2_D_3_. Compared to the NC group, cells with *Lgals3* knockdown exhibited a significant decrease in gal-3 protein level (*p* < 0.01) (Figure 7). In the NC group, the expression of NFATc1 and MMP-9 proteins were significantly inhibited by 1α,25-(OH)_2_D_3_ (*p* < 0.01). In gal-3 knockdown groups, 1α,25-(OH)_2_D_3_ had no significant effect on the expression of NFATc1 and MMP-9 proteins. These data confirmed that gal-3 contributed to OC-related protein expression regulated by 1α,25-(OH)_2_D_3_. Gal-3 knockdown significantly increased OC-related protein expression levels (*p* < 0.01) (Figure 7). These findings further suggest that gal-3 is a negative regulator of OC formation.

**Figure 8:**
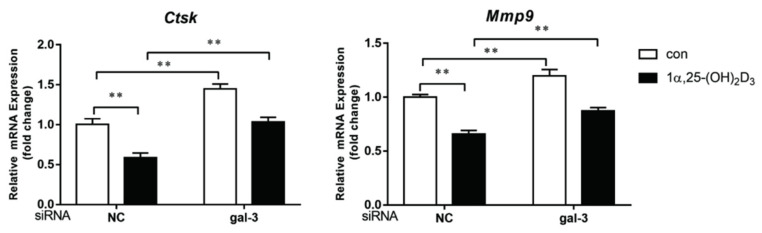
Gal-3 knockdown attenuated the inhibitory effect of 1α,25-(OH)_2_D_3_ on OC-related gene expression. Histograms show relative expression levels of genes. Data are shown as means ± SD. n = 3, ** *p* < 0.01.

mRNA expression levels of OC-related genes, *Ctsk,* and *Mmp-*9 were evaluated by qPCR on day 3 after treatment with 1α,25-(OH)_2_D_3_. In the NC group, *Ctsk* and *MMP-*9 levels were significantly inhibited by 1α,25-(OH)_2_D_3_ (*p* < 0.01). In the gal-3 knockdown groups, 1α,25-(OH)_2_D_3_ also inhibited *Ctsk* and *MMP-*9 expression. However, compared to levels in the NC groups, the inhibitory effects of 1α,25-(OH)_2_D_3_ on *Ctsk* and *MMP-*9 were significantly attenuated by gal-3 knockdown (*p* < 0.01). Additionally, gal-3 knockdown significantly increased OC-related gene expression levels (*p*< 0.01) (Figure 8). These findings were consistent with TRAP-positive OC formation and OC-related protein expression results.

**Figure 9:**
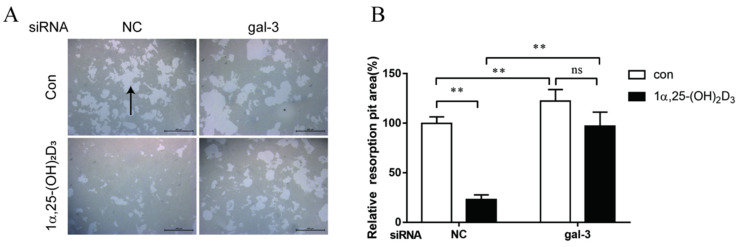
Gal-3 knockdown attenuated the inhibitory effect on bone resorption by 1α,25-(OH)_2_D_3_. (**A**) Bone resorption lacunae (marked by black arrows) observed by inverted microscopy. Bars = 400 μm. (**B**) Statistical analysis of the area of bone resorption lacunae. Data are shown as means ± SD. n = 3, ** *p* < 0.01, ns means *p* > 0.05.

To evaluate the effects of gal-3 on bone resorption regulated by 1α,25-(OH)_2_D_3_, equal number of BMMs were cultured on an osteoassay surface multiple-well plate for each group. Bone resorption lacunae were observed using an inverted microscope on day 6 after the treatment with 1α,25-(OH)_2_D_3_. We observed bone resorption lacunae in each group (Figure 9A, black arrow). Based on the area of bone resorption lacunae, in the NC group, bone resorption was significantly inhibited by 1α,25-(OH)_2_D_3_ (*p* < 0.01). In the gal-3 knockdown groups, 1α,25-(OH)_2_D_3_ had no effect on bone resorption activity. Gal-3 knockdown significantly attenuated the inhibitory effect of 1α,25-(OH)_2_D_3_ on bone resorption (*p* < 0.01). Additionally, gal-3 knockdown significantly increased OC bone resorption (*p* < 0.01) (Figure 9B). These data confirmed that gal-3 is a negative regulator of OC bone resorption and contributes to the inhibitory effect of 1α,25-(OH)_2_D_3_ on OC bone resorption.

### 2.4. Interaction between Gal-3 and VDR

**Figure 10:**
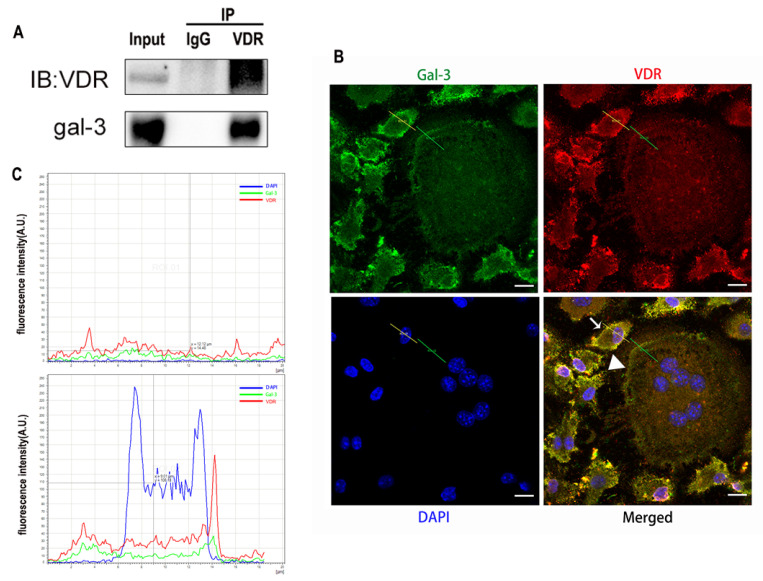
Images showing co-localization and possible interactions between gal-3 and VDR proteins. (**A**) Interaction between gal-3 and VDR proteins confirmed by co-immunoprecipitation. (**B**,**C**) Co-localization of gal-3 and VDR proteins detected by immunofluorescence. In non-merged images, gal-3 is green, VDR is red, and nuclei are blue. Bars = 10 μm.

To verify the relationship between gal-3 and VDR proteins, they were evaluated by co-immunoprecipitation and immunofluorescence double staining. The expression of VDR and gal-3 could be detected in the input group (Figure 10A). The expression of VDR and gal-3 was also detected in the protein samples precipitated by the anti-VDR antibody (Figure 10A). These results suggest that there is an interaction between gal-3 and the VDR proteins.

The expression of gal-3 (green) and VDR (red) protein was also observed by confocal fluorescence microscopy. OCPs (small cells marked by white arrows in Figure 10B) showed high expression levels of gal-3 and VDR, while gal-3 and VDR expression levels were low in OCs (large cells with multiple nuclei, marked by white triangles). Gal-3 and VDR proteins were mainly co-localized (yellow) in the cell membrane (Figure 10B). The red and green curves change in the same way, which suggests that gal-3 and VDR are co-located. (Figure 10C). These results further supported the co-localization and possible interaction between gal-3 and VDR.

## 3. Discussion

OCPs from the bloodstream migrate to the bone surface, where they can form bone-resorbing OCs under the influence of M-CSF and RANKL [26,27]. During OC formation, RANKL induces the expression of NFATc1, a master transcription factor, which then activates the TRAP gene expression [28,29,30]. The formation of OCs on the bone surface can secrete protons and cysteine proteases, leading to bone mineral dissolution and demineralized collagenous matrix degradation [31]. Ctsk, which is mainly found in OCs, is the major cysteine protease secreted by OCs [31]. In addition to Ctsk, MMP-9 is indispensable for OC migration. MMP-9 inhibitors can reduce bone resorption [27]. Therefore, Ctsk and MMP-9 are regarded as functional proteins in OCs.

Vitamin D is known as the anti-rickets vitamin, and the active form 1α,25-(OH)_2_D_3_ is involved in a variety of physiological processes. Among these physiological processes, the most important function is the regulation of calcium homeostasis together with PTH. When serum calcium is low, 1α,25-(OH)_2_D_3_ acts with PTH to increase calcium absorption from the intestine. If normal calcium cannot be maintained by intestinal calcium absorption, 1α,25-(OH)_2_D_3_ will act with PTH to increase the reabsorption of calcium from the kidney and increase calcium release from the bone stores [32]. Kitazawa et al. [33] also reported that 1α,25-(OH)_2_D_3_ increases calcium release from bone stores. They confirmed that 1α,25-(OH)_2_D_3_ can increase RANKL expression in stromal cells and support osteoclastogenesis indirectly. In addition, 1α,25-(OH)_2_D_3_ can also act directly on OCPs and conversely inhibit OC formation. Consistent with our previous results [34], we demonstrated that 1α,25-(OH)_2_D_3_ directly inhibited TRAP-positive OC formation. We also confirmed that 1α,25-(OH)_2_D_3_ inhibited the expression of OC-related genes and proteins, including Ctsk, MMP-9, and NFATc1 expression. The bone resorption activity of OCs was also inhibited by 1α,25-(OH)_2_D_3._ These data are consistent with those previous studies. For example, Sakai et al. [35] also found that 1α,25-(OH)_2_D_3_ inhibits OC formation by inhibiting the expression of c-Fos and NFATc1. Kikuta et al. [36] confirmed that 1α,25-(OH)_2_D_3_ and its analogue eldecalcitol promoted OPCs migration and inhibited OC formation by regulating the sphingosine1-phosphate (S1P) receptor system. Thus, we hypothesize that 1α,25-(OH)_2_D_3_ can both promote and inhibit OC formation under different circumstances to achieve bone health equilibrium based on previous research. Therefore, the effect of 1α,25-(OH)_2_D_3_ on bone health and its underlying mechanism should be further elucidated. In a previous study, we found that gal-3 expression changes during OC formation in the presence of 1α,25-(OH)_2_D_3_ by chance [23]. Here, we further confirmed that 1α,25-(OH)_2_D_3_ increases gal-3 expression at the protein and mRNA levels. We then used gal-3-knockdown OCPs to evaluate OC formation, bone resorption, and the expression of OC-related genes and proteins.

Gal-3 is the only member of chimera-type galectins and is structurally composed of a large N-terminal domain and one carbohydrate recognition domain (CRD). It contains three structurally distinct domains: (1) a short amino terminal consisting of 12 amino acids with a serine phosphorylation site responsible for its translocation [37]; (2) a collagen-alpha-like nearly 110 amino acid long structure rich in proline, alanine, and glycine; and (3) a C-terminal domain with nearly 140 amino acids encompassing the CRD [38]. It participates in a variety of cellular processes, such as proliferation, differentiation, migration, and apoptosis, and has a variety of biological effects, especially in nephropathy, carcinoma, and the quiescence of hematopoietic stem cells [39,40,41]. It is also an important regulator of bone remodeling. Iacobini et al. [42] found that *Lgals3*^−/−^ OBs and OCs showed impaired terminal differentiation, reduced mineralization capacity, and resorption activity. They confirmed that gal-3 is an essential factor in normal osteocyte differentiation and activity, bone reconstruction, and biomechanical balance. Simon et al. [24] proved that extracellular gal-3 inhibited OC formation and gal-3-deficient bone marrow cells displayed a higher osteoclastogenic capacity. In the present study, we also found that gal-3-knockdown OCPs showed a stronger OC formation capacity and bone resorption capacity than those of normal OCPs. The expression levels of OC-related genes and proteins, including CTSK, MMP-9, and NFATc1, increased after gal-3 knockdown. As reported previously [24], our data also suggested that gal-3 is a negative regulator of OC formation and activation. In this study, we also found that 1α,25-(OH)_2_D_3_ not only inhibited OC formation and activation but also increased gal-3 expression in mRNA and protein levels. However, the mRNA expression of gal-3 in day 5 is lower than that in day 3. This might be relative to mRNA translation and characteristic of protein expression. It has been reported that some of the gal-3 crossed the membrane and translocated into the intracellular vesicles and/or directly onto the cell surface. Furthermore, cell surface-bound and extracellular gal-3 may re-enter the cell by endocytosis and take part in a recycling loop, and is then found in the vesicular non-cytosolic compartments [43]. This may be the cause of the inconsistent expression of gal-3 protein and mRNA, which needs further investigation. When gal-3 expression was inhibited by small interfering RNAs, the inhibitory effect of 1α,25-(OH)_2_D_3_ on OC formation and activation was significantly alleviated. These data suggest that gal-3 contributes to 1α,25-(OH)_2_D_3_-mediated OC formation and activity. Immunoprecipitation and immunofluorescence assays supported the interaction between gal-3 and VDR protein, further demonstrating that gal-3 might participate in OC formation and activation via interaction with VDR induced by 1α,25-(OH)_2_D_3_. However, Nakajima et al. [22] suggested that intact gal-3 promotes OC formation, whereas cleaved gal-3 inhibits OC formation. Therefore, the role of gal-3 in the regulation of OC formation and activation by vitamin D remains to be elucidated. In any case, gal-3 is a potential new target for the prevention of bone disease and maintenance of calcium homeostasis.

## 4. Materials and Methods

### 4.1. Isolation and Culture of Osteoclast Precursors

The isolation and culture of OCPs were based on our previously reported methods with slight improvements [44]. C57BL/6 mice (5–6 weeks old) purchased from the Laboratory Animal Center of Yangzhou University were euthanized. The tibias and femurs were dislocated and the muscles were discarded. The protocol was approved by the Animal Care and Use Committee of Yangzhou University (SYXK[Su] 2017-0044, July 20th, 2017) and was carried out in accordance with the Guide for the Care and Use of Laboratory Animals of the National Research Council. The tibias and femurs were cut longitudinally. The bone cavities were flushed with serum-free α-MEM (Gibco, Carlsbad, CA, USA) until they appeared empty. A 1 mL Pasteur pipette and 200 mesh sterile screen were used to disperse any cell aggregates. The liquid was then centrifuged for 10 min at 300× *g*. The cells that deposited at the bottom of the tube were collected, and red blood cells were removed using ACK Lysing Buffer (Thermo Fisher Scientific, Asheville, NC, USA). Cells were then suspended in α-MEM supplemented with 10% fetal bovine serum (FBS) (Gibco, Carlsbad, CA, USA) and 25 ng/mL M-CSF (416-ML-050, R&D Systems, Minneapolis, MN, USA). The cells were cultured overnight in an incubator at 37 °C under 5% CO_2_. The supernatant of the culture medium was carefully collected and centrifuged at 500× *g* for 5 min to obtain BMMs. Then, BMMs were seeded in α-MEM supplemented with 10% FBS and 25 ng/mL M-CSF. They were also incubated at 37 °C in 5% CO_2_. On day 3, suspended cells were removed, and adherent cells were regarded as OCPs.

### 4.2. Cell Viability Detection by CCK-8

After the treatment with 25 ng/mL M-CSF for proliferation, 50 ng/mL RANKL (462-TEC-010, R&D Systems) and different concentrations of 1α,25-(OH)_2_D_3_ (0.1, 1, and 10 nmol/L) (D5310, Sigma-Aldrich, St. Louis, MO, USA) were added to the culture medium. Anhydrous alcohol was used as a control (0 nmol/L 1α,25-(OH)_2_D_3_). Cell viability was determined using the Cell Counting Kit-8 (CCK-8) (Dojindo, Kumamoto, Kyushu, Japan).

### 4.3. Cell Transfection

**Figure 11:**
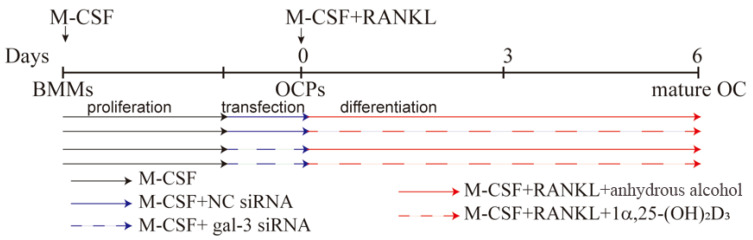
Timeline of cell transfection and the treatment with 1α,25-(OH)_2_D_3_. The black solid line represents M-CSF, the blue solid line represents M-CSF+NC siRNA, the blue dashed line represents M-CSF+gal-3 siRNA, the red solid line represents M-CSF+RANKL, and the red dashed line represents M-CSF+RANKL+1α,25-(OH)_2_D_3_.

For transfection experiments, mouse BMMs seeded in 6-well plates were pre-treated with 25 ng/mL M-CSF for 2 days. They were then transfected with gal-3 or negative control (NC) siRNA for 1 d according to the supplier’s protocol (sc-35443, 36869, Santa Cruz Biotechnology, Santa Cruz, CA, USA) in the presence of 25 ng/mL M-CSF. The end of transfection was regarded as time zero. At this point, 50 ng/mL RANKL was added to 25 ng/mL M-CSF to induce OC formation in each group. The cells were divided into four groups as follows (Figure 11): NC siRNA + anhydrous alcohol (solvent), NC siRNA + 1α,25-(OH)_2_D_3_, gal-3 siRNA + anhydrous alcohol, and gal-3 siRNA + 1α,25-(OH)_2_D_3_. The medium was changed every 2 days.

### 4.4. Formation and Identification of Osteoclasts

According to previously reported methods [45,46], 25 ng/L M-CSF and 50 ng/L RANKL were used to induce OC formation. TRAP staining and bone resorption were observed to identify OC formation. OCPs were cultured in α-MEM supplemented with 10% FBS, 25 ng/mL M-CSF, and 50 ng/mL RANKL for 6 days. The cells were fixed in 4% paraformaldehyde solution for 10 min and washed with phosphate-buffered saline (PBS). They were then stained using the TRAP Staining Kit (Sigma-Aldrich) following the manufacturer’s instructions. Cells with more than three nuclei and wine-colored granules in the cytoplasm were regarded as OCs. For bone resorption, PBS was used to repeatedly wash the plates to remove the adherent cells from Corning Osteoassay Surface Multiple Well Plates (Corning, NY, USA). Images were obtained using an inverted microscope (Leica, Wetzlar, Hessen, Germany). Image-Pro Plus (JEDA Science Technology Development, Nanjing, China) was used to calculate the bone resorption pit area.

### 4.5. Western Blotting

According to our previously described methods [46], OCs were lysed on ice for 30 min using radio immunoprecipitation assay (RIPA) lysis buffer (Applygen, Beijing, China) with protease inhibitor (Beyotime, Shanghai, China). The protein concentration in each sample was measured using the BCA Protein Assay Kit (Beyotime) and normalized. Proteins were separated by SDS-PAGE (NCM Biotech, Suzhou, China) and transferred to polyvinylidene difluoride (PVDF) membranes (Millipore, Billerica, MA, USA). Then, the PVDF membranes were blocked with 5% bovine serum albumin (BSA) (Sigma-Aldrich) at room temperature for 2 h and incubated with anti-gal-3 (Proteintech, Rosemont, IL, USA), anti-NFATc1 (Santa Cruz Biotechnology), anti-MMP-9 (Abcam, Boston, MA, USA), anti-GAPDH, and anti-β-actin (CST, Danvers, MA, USA) antibodies overnight at 4 °C. Secondary antibodies with horseradish peroxidase (Jackson Laboratories, Bar Harbor, ME, USA) were added to the PVDF, which was washed with Tris-buffered saline and Tween (TBST). Proteins were visualized using a Tanon 5200 Electrochemiluminescence (ECL) Detection System (Tanon, Shanghai, China). ImageJ (National Institute of Mental Health, Bethesda, MD, USA) was used to analyze protein expression levels.

### 4.6. Quantitative Real-Time Polymerase Chain Reaction (qPCR)

RNA was extracted by TRIzol reagent (Invitrogen, Carlsbad, CA, USA). cDNA was synthesized using HiScript QRT SuperMix (Vazyme, Nanjing, China), and then qPCR was conducted using ChamQ SYBR qPCR Master Mix (Vazyme) according to the manufacturer’s instructions. Primer sequences were designed based on *Ctsk*, *MMP-*9, *Lgals3,* and *Gapdh* gene sequences searched from NCBI of mouse and are shown in Table 1. *Gapdh* expression was used as an internal control. The cycle reaction was set at 95 °C for 10 s and 60 °C for 30 s for 40 cycles.

### 4.7. Co-immunoprecipitation

Co-immunoprecipitation studies were performed using previously described methods, with slight modifications [47]. Extracts of about 200 μg of protein per sample were precleared with protein A/G beads and then mixed with 2 μg of VDR antibody or control non-immune rabbit Ig immobilized on protein A/G beads in an “immunoprecipitation buffer” supplemented with protease inhibitors. The reactions were then incubated overnight at 4 °C. After being washed three times with 0.1% PBS-Tween, proteins degenerated by SDS buffer were resolved by SDS-PAGE and transferred onto PVDF membranes. The Western blotting was then probed with antibodies against gal-3 at 4 °C overnight.

### 4.8. Immunofluorescent Staining of VDR and Gal-3

OCs cultured on sterile coverslips were washed with PBS. They were fixed with a 4% paraformaldehyde solution. Triton X-100 (0.5%, Amresco, Solon, OH, USA) was used for membrane permeabilization at room temperature. Cells were blocked with 5% BSA and incubated with anti-gal-3 and anti-VDR antibodies overnight at 4 °C. An IgG negative control was set. Phalloidin-iFluor 555 Reagent (Abcam) was used to detect F-actin, and nuclei were visualized with DAPI (Beyotime) according to the manufacturer’s instructions. Imaging was performed using a fluorescence microscope (Leica DMI3000B; Wetzlar). Immunofluorescent co-located figures were analyzed by Leica software, and a line segment from the top left to the bottom right (draw a line from the top left to the bottom right crossing the interested area). The point in the upper left is the origin, the *x*-axis represents the distance from the origin, and the *y*-axis represents the intensity of fluorescence. The green line across OCs which fluorescence intensity presented in the Figure 6B first figure, and the yellow line across OCPs which fluorescence intensity presents in the Figure 6B second figure. The red curve represents VDR, the green curve represents gal-3, and the blue curve represents the nucleus.

### 4.9. Statistical Analysis

Statistical analysis of all data was performed using GraphPad Prism 7 (GraphPad Software). Each experiment was repeated at least three times in vitro. Statistical significance was defined as *p* < 0.05.

## 5. Conclusions

**Figure 12:**
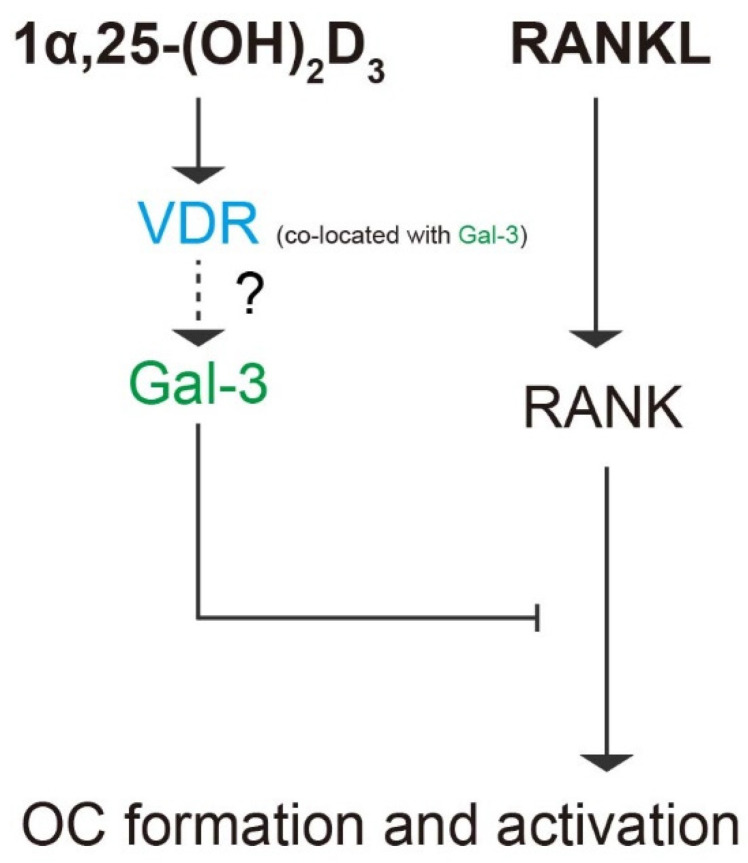
Schematic diagram of the role of gal-3 in OC formation and activation regulated by 1α,25-(OH)_2_D_3_. Briefly, 1α,25-(OH)_2_D_3_ inhibited OC formation and activation induced by RANKL and increased gal-3 expression. Gal-3, a negative regulator of OC formation and activation, colocalized with VDR and contributed to the inhibitory effect of 1α,25-(OH)_2_D_3_ on OC formation and activation.

In our study, we confirmed the regulatory effects of the 1α,25-(OH)_2_D_3_/VDR/gal-3 axis on the osteoclastogenesis potential of BMMs. OC formation and activation induced by RANKL via gal-3 was inhibited by 1α,25-(OH)_2_D_3_, which is a negative regulator of OC formation and activation. Figure 12 provides a visual overview of the detailed mechanism underlying OC formation and activation revealed in our study.

## Figures and Tables

**Table 1:** Sequences of PCR primers used in this study.

Genes	Sequences	
*Lgals3*	Forward (5′-3′)	GTACAGCTAGCGGAGCGG
	Reverse(5′-3′)	CGGATATCCTTGAGGGTTTG
*Ctsk*	Forward (5′-3′)	CGCCTGCGGCATTACCAA
	Reverse(5′-3′)	TAGCATCGCTGCGTCCCT
*Mmp-*9	Forward (5′-3′)	GCCCTGGAACTCACACGACA
	Reverse(5′-3′)	TTGGAAACTCACACGCCAGAAG
*Gapdh*	Forward (5′-3′)	AAATGGTGAAGGTCGGTGTG
	Reverse(5′-3′)	TGAAGGGGTCGTTGATGG

## Data Availability

All relevant data are presented in the manuscript. Raw data are available upon request from the corresponding author.

## Abbreviations

OCs	Osteoclasts
RANKL	Receptor activator of NF-kB ligand
M-CSF	Macrophage colony-stimulating factor
TRAP	Tartrate-resistant acid phosphate
MMP-9	Matrix metalloproteinase-9
NFATc1	Nuclear factor of activated T cells cytoplasmic 1
Ctsk	Cathepsin K
BMMs	Bone marrow-derived monocytes
OBs	Osteoblasts
OCPs	Osteoclast precursors
VDR	Vitamin D receptor
